# Cladoceran density and age structure is highly variable through time in three ponds in Northwest Indiana

**DOI:** 10.17912/micropub.biology.001912

**Published:** 2026-03-13

**Authors:** Fabiola M. Fontán-Fontán, Kacie L. Jonasen, Abigail M. Merrick, Catherine L. Searle

**Affiliations:** 1 Department of Biological Sciences, Purdue University West Lafayette, West Lafayette, Indiana, United States; 2 Departamento de Biología, University of Puerto Rico at Humacao, Humacao, Humacao, Puerto Rico

## Abstract

Zooplankton are important primary consumers in freshwater food webs. We aimed to identify common zooplankton genera and describe population dynamics through time in three ponds. We measured the presence, density, and age structure of zooplankton over three years (2016-2018) from spring to fall. We found three common genera in all ponds: *Daphnia* , *Ceriodaphnia* , and *Simocephalus* . The presence, density, and age structure of all three genera was highly variable through time. Ephemeral ponds had higher densities in 2017 when rainfall was higher than other sampled years. This work adds to the understanding of zooplankton population dynamics in small ponds.

**Figure 1. Data from three common Cladocera genera at our three sites:**
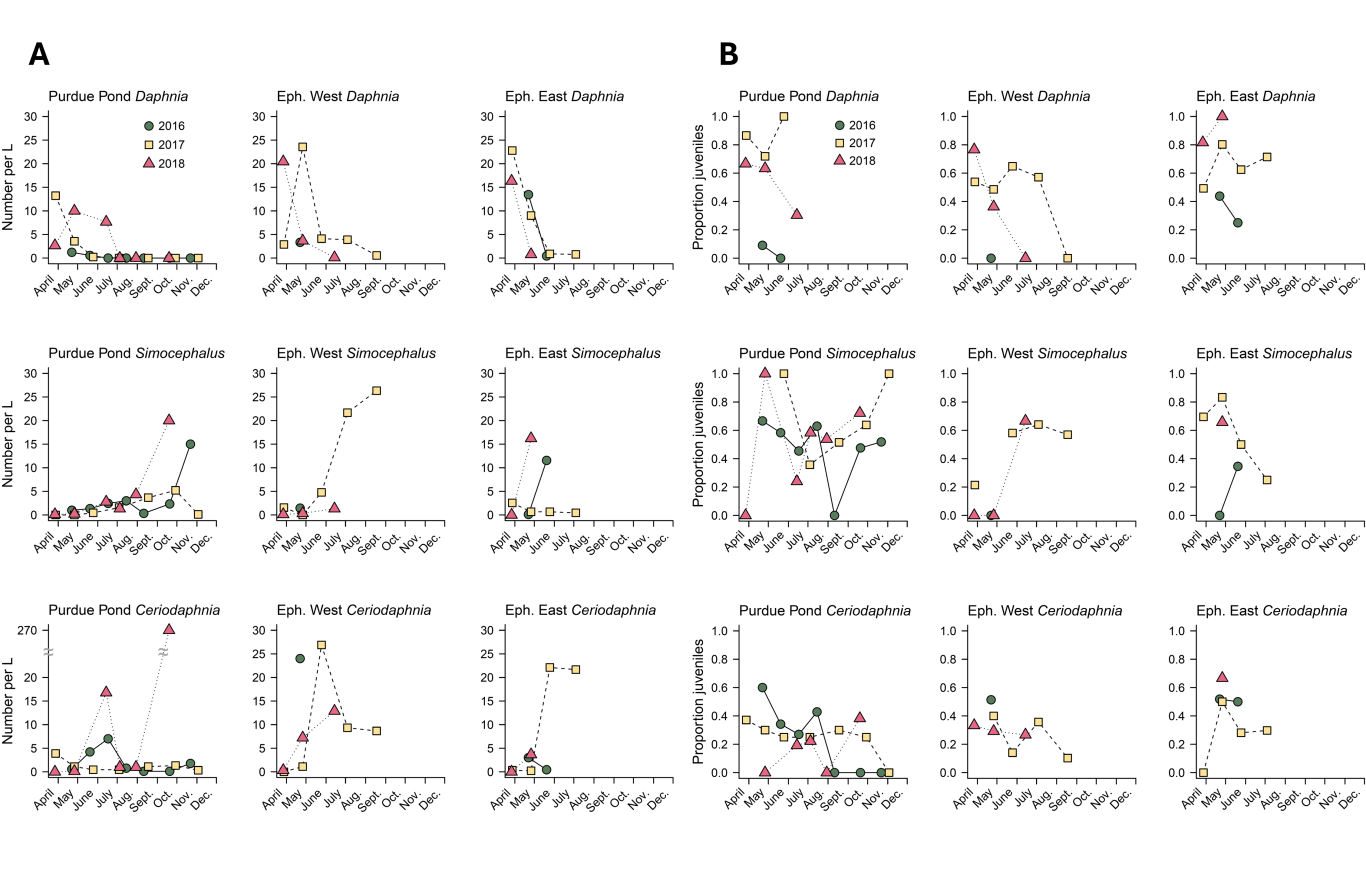
Data from three common Cladocera genera at our three sites showing A) density and B) proportion of juveniles. The top row shows densities for *Daphnia* , middle row for *Simocephalus* , and bottom row for *Ceriodaphnia* . Ponds are shown on each panel with Purdue Pond on the left, Ephemeral West (“Eph. West”) in the middle, and Ephemeral East (“Eph. East”) on the right. Each year is designated as a different shape with 2016 as green circles, 2017 as yellow squares, and 2018 as red triangles. On the x-axis, the tick locations represent the 15 ^th^ day of each month. In panel A, all sampling times when water was present are included (i.e., values of zero indicate that water was present, but individuals of that genus were not present in our water sample). Note the y-axis break for Purdue Pond *Ceriodaphnia* in panel A to accommodate one very high density in 2018. In panel B, all sampling times where individuals were present are included (i.e., a value of zero indicates that individuals of that genus were present in our sample, but all were adults). &nbsp; &nbsp;

## Description

Zooplankton are a key component of freshwater food webs and can be indicators of ecosystem health (Gannon & Stemberger 1978; Jeppesen et al. 2011). Many species of zooplankton filter feed in their role as primary consumers and their presence can improve water clarity (Higgins et al. 2014; Bess et al. 2021). Additionally, zooplankton can be substantial sources of food for many freshwater organisms ( *e.g* ., Anton-Pardo & Adámek 2015; Hädicke et al. 2017). The impacts of freshwater zooplankton on communities and ecosystems can be mediated by their species, density, and age structure (e.g., Bess et al. 2021, Rudolf & Rasmussen 2013). Thus, characterizing zooplankton populations is essential for understanding and managing freshwater systems.

Zooplankton density and age structure can change with predator abundance, primary production, the presence of conspecifics, and water chemistry (Arnott & Vanni 1993; Feuchtmayr et al. 2010; Searle et al. 2016; Searle et al. 2018; Blackwood et al. 2024). Because many of these abiotic and biotic conditions change seasonally, there can be large variation in zooplankton density and abundance throughout a given year (Olmo et al. 2012; Salman et al. 2014). Additionally, hydroperiod can affect the composition, abundance, and size structure of zooplankton communities and populations (Girdner & Larson 1995; Serrano & Fahd 2005; Drenner et al. 2009).

To understand how the presence, density, and age structure of zooplankton taxa vary through time, we monitored zooplankton communities in three ponds for three consecutive years. We focused on the order Cladocera because it is an essential component of aquatic food webs and several cladoceran genera are considered model organisms for studying ecology and evolution (Seda & Petrusek 2011). We aimed to answer the following questions: 1) What are the common Cladocera genera present in these ponds? 2) Does the presence and density of the common taxa vary consistently across years and months? 3) Does cladoceran age structure vary across years and months?

To answer these questions, we sampled three ponds in Tippecanoe County, Indiana, USA, every month from spring through fall for three years. At each sampling date, we quantified the density of the common Cladocera in our samples along with the age structure (i.e., proportion of each species that were juveniles). Overall, we found that the presence, density, and age structure of three common Cladocera genera in our study were highly variable across dates and years. Our statistical models showed that both year and date were significant predictors for some response variables, but only for some species. In all, the high variability in the presence, density, and age structure of our three focal taxa make it difficult to identify consistent patterns across years and months.

Our first question was: what are the common Cladocera genera present in these ponds? We found three common genera of Cladocera at our ponds: *Daphnia* ( *D. pulex* and *D. dubia* ), *Simocephalus* (primarily *S. serrulatus* ), and *Ceriodaphnia* (primarily *C. reticulata* ). Individuals from each genus were present in all ponds at least once each year. We did not observe a single, dominant genus in any pond across months or years; there was high variation in the density and presence of each genus through time ( Figure 1A ). These three genera are relatively common throughout ponds and lakes in North America, with multiple species present within each genus (Gillooly and Dodson 2000, Dodson et al. 2010). Because we were only able to sample one region of the permanent pond (Purdue Pond), we may have missed some genera that occurred in other locations within this site. However, the same three dominant genera were present in all sites despite differences in hydroperiod (i.e., Purdue Pond is permanent while the other two sites are ephemeral).

We then asked: does the presence and density of the common taxa vary consistently across years and months? The only significant predictor of density was “date” for *Daphnia* (F _1,56_ = 29.27, *p* < 0.001, β = -0.45 ± 0.08; Figure 1A ). Density was not explained by date or year in any other genera ( *p* > 0.1 for all others). Both year and date were significant predictors for the presence of *Daphnia* (year: *X ^2^* (2) = 11.94, *p* = 0.003, 2017: β = 5.69 ± 2.43, 2018: β = 1.94 ± 1.53; date: *X ^2^* (1) = 56.73, *p* < 0.001, β = -0.09 ± 0.03). For both *Simocephalus* and *Ceriodaphnia,* date was a predictor for presence ( *Simocephalus* : *X ^2^* (1) = 7.93, *p* = 0.005, β = -0.01 ± 0.001; *Ceriodaphnia* : *X ^2^* (1) = 11.83, *p* < 0.001, β = -0.02 ± 0.01), but not year ( *Simocephalus* : *X ^2^* (2) = 1.16, *p* = 0.56, *Ceriodaphnia* : *X ^2^* (2) = 5.82, *p* = 0.054). To summarize, the only significant predictor in our density models was date for *Daphnia* , where the density of *Daphnia* declined throughout the year. In contrast, date was a significant predictor for the presence all three taxa, where their presence decreased as the year progressed, driven by the drying ponds. Thus, throughout the spring-fall seasons, the presence of each zooplankton genus changed in a predictable manner, while the density experienced either little or unpredictable change through time.

When we considered maximum densities, both of our ephemeral ponds experienced maximum densities of most taxa in 2017, when rainfall was higher and the hydroperiod longer than the two other years (Table 1). Specifically, the maximum density of *Daphnia* occurred in 2017 for all three ponds, the maximum density of *Simocephalus* occurred in 2018 for Purdue Pond and Ephemeral East and in 2017 for Ephemeral West, and the maximum density of *Ceriodaphnia* occurred in 2017 for the two ephemeral ponds and in 2018 for Purdue Pond. In contrast, none of the ponds or genera experienced maximum densities in 2016 when rainfall was lowest. Thus, maximum zooplankton density may be limited by yearly rainfall, especially in ephemeral ponds. As climate change creates hotter summers for Indiana (Widhalm et al. 2018), rainfall may decrease and ephemeral ponds may experience faster drying times, which could lead to lower maximum densities, causing numerous changes to the aquatic food web.

Finally, we asked: does cladoceran age structure vary across years and months? We found that the impact of year and date on age structure varied depending on the genus. Year was a significant predictor for the proportion of *Daphnia* that were juveniles ( *X ^2^* (2) = 11.51, *p* = 0.003, 2017: β = 36.18 ± 647.64, 2018: β = 35.07 ± 647.63; Figure 1B ) but not date ( *X ^2^* (1) = 2.28, *p* = 0.131). *Daphnia* populations had a higher proportion of juveniles in both 2017 and 2018 compared to 2016. Neither year nor date was a predictor for the proportion of *Simocephalus* that were juveniles (year: *X ^2^* (2) = 2.41, *p* = 0.300; date: *X ^2^* (1) = 3.38, *p* = 0.066: Figure 1B ). In contrast, both year and date were predictors for the proportion of *Ceriodaphnia* that were juveniles (year: *X ^2^* (2) = 9.60, *p* = 0.008, 2017: β = -4.91 ± 1.73, 2018: β = -3.00 ± 2.03; date: *X ^2^* (1) = 9.05, *p* = 0.003, β = -0.08 ± 0.05; Figure 1B ). The year effect in *Ceriodaphnia* , was in the opposite direction as *Daphnia* ; there was a higher proportion of juveniles in *Ceriodaphnia* in 2016 compared to the two other years. Thus, different species of zooplankton varied demographically through time in different ways.

Results from our study add to the understanding of zooplankton community composition across time in small ponds. Our results show that the presence, density, and age structure of cladocerans can be highly variable across time, and future studies are necessary to understand how these temporal changes to cladoceran populations affect other members of the community ( *e.g.,* fish, phytoplankton). There are many biotic and abiotic components of these ponds that may have caused the observed variation across seasons and years, including the presence of predators, nutrient availability, temperature, water depth, and water chemistry ( *e.g* ., Arnott & Vanni 1993; Serrano & Fahd 2005; Feuchtmayr et al. 2010; Searle et al. 2016). Future studies should measure and investigate how these variables change through time and affect zooplankton populations and communities. Additionally, future studies that measure fine-scale changes to zooplankton communities (e.g., at the species level or within pond microhabitats) would likely reveal additional temporal patterns that may have broad ecological significance. Overall, quantifying the variability of zooplankton communities can help scientists better understand and manage freshwater ecosystems.

## Methods

Our study sites were three ponds at the Purdue Wildlife Area in Tippecanoe County, Indiana. One of the ponds (Purdue Pond; 40º27’14” N, 87º03’20” W) is a large wetland that varies in size throughout the year but does not dry completely. The two other ponds (Ephemeral West; 40º27’08” N, 87º03’18” W, and Ephemeral East; 40º27’03” N, 87º03’08” W) are small, ephemeral ponds that typically only contain water in the spring and early summer.

We sampled each pond approximately once a month from spring to fall for three years (2016-2018; Table 1). We visited all three ponds at each sampling date, but only collected samples if there was at least 15 cm of water present in a pond, which was necessary to implement our sampling methods. At each pond with sufficient water, we collected 3L of pond water at three locations (9L total per pond) using a 1L scoop placed just below the surface of the water. The water was passed through a 333um mesh filter to concentrate the zooplankton and brought back to the laboratory for enumeration. This mesh size is small enough to capture the majority of cladoceran age classes but may fail to capture juveniles of some of the smaller species (Lynch 1980). Thus, it is possible that the mesh size we used allowed very small *Ceriodaphnia* to pass through (Lynch 1980), but because we still observed a high number of juvenile *Ceriodaphnia* in our samples we expect that the impact of these potential omissions was minimal. We used a Ward counting wheel (Wildco) and a stereomicroscope to count the number of individuals in each genus of Cladocera in each sample (identified using Dodson et al. 2010 and Haney et al. 2013). We also classified each individual as juvenile or adult based on size and the width of the brood chamber in females; juveniles have a flat brood chamber while adults have a wide brood chamber. We confirmed that this method of distinguishing adults from juveniles was accurate by scanning samples to determine the size of females carrying eggs and confirming that individuals we classified as juveniles were below this general size (as per Duffy & Hall 2008). We then converted our count data into density of each genus in the original water sample in number per liter. We used density values to calculate presence/absence and considered a genus to be absent if we were unable to sample a pond due to lack of water or if we did not find any individuals from that genus in our sample; thus, absence indicates that a genus was absent at a site for any reason. We calculated the total rainfall for each year from April to November using data from West Lafayette, Indiana, USA, which is approximately 13 km from our study sites (NOAA, NCDC 2018).

**Table 1** : Information on our three ponds. Below each range of dates is the total number of successful sampling events for each pond each year (i.e., dates where we visited the pond and there was at least 15cm of water). For the two ephemeral ponds, we also list the first date where we observed the pond to be dry; pond drying could have occurred any time between the last successful sampling date and the “first observed dry” date. Rainfall was calculated based on monthly rainfall from April to November in West Lafayette, IN, USA (NOAA, NCDC 2018).

&nbsp;

**Table:** 

Year	Purdue Pond (# sampling dates)	Ephemeral West (# sampling dates; first observed dry)	Ephemeral East (# sampling dates; first observed dry)	Rainfall (cm; April –November)
2016	5 May – 4 November (7)	5 May (1; 2 June)	5 May – 2 June (2; 30 June)	72.52
2017	11 April – 17 November (7)	11 April – 1 September (5; 13 October)	11 April – 18 July (4; 1 September)	99.11
2018	10 April – 3 October (6)	10 April – 28 June (3; 19 July)	10 April – 10 May (2; 28 June)	82.83

&nbsp;

All analyses were conducted in R (version 4.1.0; R Core Team 2021). For all models, our response variables were year (factor, with 2016 as reference year) and date (day of year) as fixed effects and pond as a random effect. We did not directly analyze the effects of rainfall, but annual rainfall varied across the three years (Table 1). We analyzed density using a linear mixed effects model ( *lmerTest* package; Kuznetsova et al. 2017). We did not statistically analyze our results for maximum density due to lack of statistical power and replication. We constructed generalized linear mixed effects models for presence/absence of each genus with a binomial link function ( *lme4* package; Bates et al. 2015). We also examined the age structure by calculating the percent of each genus made of juveniles at each time point and used binomial generalized linear mixed effects models ( *lme4* package; Bates et al. 2015). In our model for percent juveniles, we omitted values of zero that were caused by lack of data (i.e., when water was not present or when the species was not observed in a sample). For all response variables we constructed separate models for each species and corrected for multiple comparisons using a Bonferroni correction. Our qualitative results for density, presence/absence, and age structure were the same whether we used our Bonferroni-corrected α = 0.0167 (for three tests for each response variable) or the more traditional α = 0.05.
